# Adolescent caffeine use and problematic school behavior: A longitudinal analysis of student survey data and teacher observations

**DOI:** 10.1002/jad.12383

**Published:** 2024-07-25

**Authors:** Alfgeir L. Kristjansson, Christa L. Lilly, Michael J. Mann, Megan L. Smith, Steven M. Kogan, Hannah M. Layman, Jack E. James

**Affiliations:** ^1^ Department of Social and Behavioral Sciences West Virginia University School of Public Health, West Virginia Prevention Research Center Morgantown West Virginia USA; ^2^ Department of Epidemiology and Biostatistics West Virginia University School of Public Health Morgantown West Virginia USA; ^3^ Department of Community and Environmental Health Boise State College of Health Sciences Boise Idaho USA; ^4^ Department of Human Development and Family Science University of Georgia College of Family and Consumer Sciences Athens Georgia USA; ^5^ Department of Psychology Reykjavik University Reykjavik Iceland

**Keywords:** caffeine, middle school, problem behavior, self‐control, Young Mountaineer Health Study

## Abstract

**Introduction:**

Caffeine is a psychostimulant possessing arousal, motor activation, and reinforcing properties, which is consumed daily by most adolescents aged 12–19 years. Although current understanding of the implications of adolescent caffeine consumption for school behaviors remains incomplete, studies have shown that in addition to acute effects of the drug, in common with other habit‐forming psychoactive substances, regular use leads to physical dependence, evidenced by recurring negative withdrawal symptoms.

**Methods:**

Employing two waves of longitudinal data, we tested the prospective association between daily caffeine use and homeroom teacher‐observed self‐control and problem behavior in a sample of middle‐school students in 20 schools in West Virginia in the United States. Caffeine was operationalized with two dichotomized variables, daily consumption of <100 mg, and daily consumption of >100 mg, versus no daily use. Gender, mother's education, family financial status, social support by primary caregiver and adults in school, and school climate, were applied as covariates in linear mixed models.

**Results:**

Daily caffeine use of >100 mg was robustly and inversely associated with self‐control and positively associated with problem behavior.

**Conclusions:**

Caffeine consumption and associated withdrawal symptoms may be an important factor in problematic school behavior among adolescents. Recent advent of highly concentrated caffeine products (e.g., caffeine “shots”) commonly marketed directly at youth, should give rise to concerns including consideration about limiting caffeine consumption among children and youth.

The psychostimulant effects of caffeine include subjective arousal, psychomotor activation, and reinforcing properties (Ferré, 2016), which are believed to be due primarily to competitive blockade of centrally‐ and peripherally located adenosine receptors (Dunwiddie & Masino, 2001). The *Diagnostic and Statistical Manual of Mental Disorders*, *5th edition* (American Psychiatric Association, 2013; *DSM‐5*) lists several caffeine‐related diagnoses, including “caffeine intoxication,” “caffeine withdrawal,” “other caffeine‐induced disorders” (e.g., “caffeine‐induced anxiety disorder” and “caffeine‐induced sleep disorder”), and “unspecified caffeine‐related disorder.” In common with other psychoactive substances, regular caffeine consumption leads to physical dependence, evidenced by abstinence‐induced symptoms of withdrawal, including headache, sleepiness, lethargy, mood disturbance, and flu‐like symptoms (Juliano & Griffiths, 2004). Withdrawal effects typically begin within 12–24 h after abstinence and may persist for 2–9 days. Cessation of as little as 100 mg per day (similar to a single cup of filter coffee or a typical eight‐fluid ounce/237 milliliter can of a widely consumed energy drink) and less can produce symptoms (e.g., Lieberman et al., 1987; Smit & Rogers, 2000). Moreover, studies show that decreases in psychomotor performance (not necessarily discernible to the individual) are detectable after as little as 6–8 h since caffeine was last ingested (Heatherley et al., 2005).

Most youth aged 12–19 years consume caffeine daily (James et al., 2015; Kristjansson et al., 2013). Moreover, notwithstanding earlier evidence of relatively stable levels of intake over time (Drewnowski & Rehm, 2016; Temple, 2009, 2019), more recent evidence suggests that overall caffeine consumption by youth may be trending upwards. Specifically, recent studies have reported the majority of youth consuming >100 mg of caffeine per day (Kristjansson, Santilli, et al., 2023, 2022), a level of intake sufficient to produce recurring cycles of withdrawal effects (Temple, 2019). Additionally, caffeine use among middle‐ and high‐school students has been found to be positively related to depressive symptoms (Luebbe & Bell, 2009) and physical complaints such as stomach aches and headaches (Kristjansson et al., 2014), and inversely related to sleep duration and quality (Halldorsson et al., 2021; James et al., 2011), most of which are categorized as withdrawal symptoms of caffeine ingestion. Hence, studies have repeatedly documented caffeine withdrawal symptoms among young regular users (Temple, 2009, 2019). Caffeine consumption among adolescents has also been shown to be positively associated with angry mood (James et al., 2015) and aggressive and/or violent behaviors, in both cross‐sectional and longitudinal studies (Kristjansson et al., 2013). Furthermore, caffeine consumption among youth aged 12–19 years has been found to be both cross‐sectionally and longitudinally related to other substance use, such as alcohol, including age of onset and quantity consumed (Kristjansson et al., 2018; Mann et al., 2016); binge drinking (Kristjansson et al., 2015); onset of nicotine, tobacco, and e‐cigarette use (Kristjansson et al., 2018); and illicit drug use (Butler et al., 2019; Terry‐McElrath et al., 2014). Notably, despite the potential for diverse negative effects, caffeine is distinguished by being the only psychoactive substance that is readily available to, and widely marketed directly to, children and youth (Pomeranz, 2012).

As a psychoactive substance with addictive properties, caffeine consumption patterns are typically established soon after regular use is initiated, whether initiation occurs during adulthood or childhood (Temple, 2019). Historically, caffeine consumption among children and youth was largely confined to cola drinks and other caffeinated soda, which generally contain between 20 and 40 mg of caffeine per eight‐fluid ounces/237 mL serving (Temple, 2009, 2019), compared to a typical cup of coffee which contains between 80 and 120 mg of caffeine (James, 1997). However, the nature of caffeine consumption among youth has changed considerably during the recent past. Caffeine is now more commonly ingested by youth in fewer but larger doses, such as via energy drinks (Tran et al., 2016), many of which include concentrations of caffeine similar to regular coffee (Temple, 2009, 2019). The concentration of caffeine in energy drinks may be as high as 30 mg per fluid ounce/30 mL, with such drinks commonly packaged in 8–12 fluid ounce containers (237 mL/355 mL) and sometimes in even larger containers such as in 17 fluid ounce/500 mL cans. This, in turn, raises concerns about potential negative health and behavioral impacts of higher levels of acute consumption, irrespective of whether the total number of doses consumed per day may not have changed appreciably.

## 1 THE CURRENT STUDY

Despite the now considerable body of evidence of caffeine‐associated negative behavioral and psychosocial effects among children and youth, substantial details of relevant mechanisms remain to be elucidated. It is likely that competitive blockade of adenosine receptors is at least partially responsible for some outcomes, such as increased arousal, anxiety, and disturbed sleep. The causal pathways for other effects, such as depression, may be less direct, wherein the development of depressive mood may be one outcome of chronic caffeine‐induced sleep disruption. Additionally, acute effects of caffeine ingestion and symptoms of caffeine withdrawal may separately or together exhibit shared variation with personality dispositions such as risk‐taking and/or sensation‐seeking. Thus, without aiming to directly elucidate specific causal pathways, the present study assumes that improved knowledge of the array of caffeine's associated negative behavioral and psychosocial effects will in due course contribute to better understanding of relevant causal mechanisms. In particular, considering recently documented changes in caffeine consumption patterns among children and youth and previously observed relationships between caffeine use and negative behavioral effects including symptoms of withdrawal, important questions concern whether caffeine may negatively impact school‐based behavior, well‐being, and thus future prospects among youth. A previous cross‐sectional study found an inverse association between adolescent caffeine use and academic achievement, with the relationship being partly mediated by daytime sleepiness (James et al., 2011). Building upon those findings, the aim of the present study was to assess the longitudinal relationship between caffeine use and teacher‐observed behavior of pupils in school, with particular reference to self‐control and classroom problem behavior.

## 2 MATERIALS AND METHODS

### 2.1 Sample

The present analyses are based on Waves 1 and 3 from the Young Mountaineer Health Study (YMHS) cohort (Kristjansson, Santilli, et al., 2022) conducted about 12 months apart. Between the fall of 2020 and spring of 2023, a single cohort of students enrolled in 20 geographically diverse public middle schools in five counties in West Virginia were followed six times (twice per year) from Grades 6 through 8. During the fall of 2020 and 2021, along with student survey Waves 1 and 3, homeroom teachers also completed individual student behavior checklists. These data (Waves 1 and 3 of student survey data and teacher report data collected at the same time) were merged using a unique individual research ID number and applied in the analyses for this paper.

During student survey Wave 1 assessment from October to December 2020, 1671 students attended regular school in either face‐to‐face or hybrid formats (part in person, part virtual) with the variation being due to COVID‐19 pandemic mitigation efforts at the time. Not included were participants who attended a virtual‐only format, which in West Virginia was available only briefly as a limited option during the height of the COVID‐19 pandemic. Of the students attending some amount of in‐person school, 1348 (80.7%) completed the Wave 1 survey (mean age of 11.5 years). For student survey Wave 3 that was conducted between October and December 2021 using identical data collection protocols, 1908 of 2296 registered students (83.1%) completed the Wave 3 survey. Homeroom teacher reports were completed for all students and linked with individual student survey responses via the aforementioned research IDs. In sum, for the present analysis, 1348 student surveys and 1645 teacher reports were collected in Wave 1, and 1908 student surveys and 2026 teacher reports in Wave 3, 12 months later.

### 2.2 Procedure

The Institutional Review Board of West Virginia University approved all study protocols (#1903499093A001). A detailed protocol manuscript has been published elsewhere (Kristjansson, Santilli, et al., 2022). The YMHS research team consists of investigative members, project manager, three county data collection leaders, and 20 supervising contact agents (one in each school), to implement the total data‐collection effort. All five county Superintendents and 20 school Principals provided approval for participation in the study. Survey data collection was confidential, and protocols ensured anonymity of participants. No identifying information was collected. An introductory letter and/or email was sent to all parents and caregivers before each survey wave to notify them about the study and provide an opportunity to opt their children out of participation and to raise questions or concerns they may have had about the study. Consent procedures were explained, and participants informed about their rights to decline participation without repercussions. Data collection procedures utilized an honest‐broker system to link individual data across study waves while ensuring individual confidentiality (Kristjansson, Santilli, et al., 2022). Students responded to the confidential computer‐based surveys using Qualtrics software with the device camera turned on, either in school or during designated online classes from home, depending on accessibility during COVID‐19 mitigation efforts at each time point. The initial screen of the survey included a consent description and explained that continuing the survey indicated verbal consent to participate. Data collection was supervised by research staff. Students were compensated with a small token for responding to the survey in each wave (e.g., a pencil with the study logo and a Kind bar) and invited to enter a drawing for an iPad of which one was drawn in each survey wave. Homeroom teacher reports were collected via paper‐and‐pencil survey form and sealed in blank envelopes upon completion for each individual student. Research staff, who do not have access to study data files, entered the teacher report data into a spreadsheet, which was subsequently merged with the main data file by the study principal investigator/principal author. Teachers were compensated with a $10 gift card per each student response they provided.

### 2.3 Measures dependent variables

The two study‐dependent measures were home‐room teacher reports of two different features of student behavior.

*Problem behavior* was measured with the 18‐item brief problem monitor, an abbreviated form of the Child Behavior Checklist (Piper et al., 2014). Example items include “Describe the student within the last 30 days” in terms of the following: “Can't sit still; restless or hyperactive” and “Self‐conscious or easily embarrassed.” Item scores ranged from 0 = “Not true,” 1 = “Somewhat true,” and 2 = “Very true.” Scores were summed to form a scale with a possible range of 0–36 (*α* = .91 Wave 1, .92 Wave 3).

*Self‐control* was measured with a 15‐item scale developed by (Humphrey, 1982). Example items include, “How often does this student;” “Work towards a goal” and “Make careless mistakes because he/she rushes through work.” Items scores ranged from 1 = “Never” to 5 = “Almost always.” Scores were summed to form a scale with a possible range of 15–75 (*α* = .92, both time points). Scores for the Problem behavior and Self‐control measures were summed even if individual items were omitted, such that lower values were possible for both scales.

### 2.4 Independent variables

#### 2.4.1 Caffeine use

The caffeine measure was designed to assess daily consumption from multiple types of beverages. This inventory has been validated in multiple publications (James et al., 2011; Kristjansson et al., 2013, 2023). Respondents were asked, “How many cups/glasses/cans/or bottles do you usually drink each day of the following drinks?” “Coffee”, “Tea”, “Caffeinated soda (e.g., cola drinks, Mountain Dew, Dr. Pepper)”, and “Energy drinks that contain caffeine (e.g., Red Bull, Monster, Rockstar, Bolt, etc.)”. Response options ranged from 1 = “None” to 7 = “6 or more (glasses/cups/cans/bottles).” Participants were also asked: “How many caffeine ‘shots’ (e.g., *5‐h Energy*) do you usually have each day?” and scored on the same scale as the previous four caffeine beverage questions. Each type of caffeine beverage was then weighted to reflect approximate proportional differences in caffeine content (Kristjansson, Mann, et al., 2022; Tanda & Goldberg, 2000): coffee (6×), tea (3×), soda/pop (1×), energy drinks (3×), and caffeine shots (12×). A continuous variable was created by summing the weighted responses for all five caffeine questions and converting them to milligrams per day. The resulting measure of estimated daily caffeine consumption was operationalized as three factors: 0 = “no caffeine use”, 1 = “daily usage of <100 mg/day,” and 2 = “daily usage of 100+ mg/day.” The three factors were then operationalized as two 0/1 coded variables with “no caffeine use” as the common referent group.

*School connectedness* was measured with a five‐item scale developed by (Resnick, 1997). Sample items include “I feel close to people at this school” and “I feel like I am a part of this school.” Item scores ranged from 1 = “Strongly disagree” to 5 = “Strongly agree.” Scores were summed to form a scale with a range of 5–25 (*α* = .79 Wave 1, .85 Wave 3).

#### 2.4.2 Supportive adults in school

Support from adults in school was measured with a four‐item School as a Protective Factor Scale (Mann et al., 2024). Sample items include “The adults at my school care about me,” and “The adults at my school are fair and kind to me.” Item scores ranged from 1 = “Strongly disagree” to 5 = “Strongly agree.” Scores were averaged to form a scale with a range of 1–5 (*α* = .84 Wave 1, .87 Wave 3).

*Primary caregiver support* was measured in two steps. First, respondents were asked to select from a 13‐item drop‐down menu who is their primary caregiver (e.g., mother, father, stepmother, stepfather, etc.). Via computerized skip logic, the survey would then automatically refer to the primary caregiver in the 5‐item parental behavior inventory originally developed by (Schaefer, 1965). Sample items include “My [‎primary caregiver] is able to make me feel better when I am upset,” and “My [primary caregiver] is easy to talk to.” Item scores ranged from 1 = “Not like,” 2 = “Somewhat like,” and 3 = “a lot like” and were summed to form a scale with a range of 5–15 (*α* = .87 Wave 1, .91 Wave 3).

#### 2.4.3 Demographic control variables

Student gender was measured with the question: “How do you describe your gender?” and the following four categories: 1 = “Boy,” 2 = “Girl,” 3 = “Gender Non‐conforming,” 4 = “Other (Please specify).” Due to low frequencies, Groups 3 and 4 were merged as “other gender.” Family structure was assessed with a multiple‐response string of questions pertaining to “Who lives in your household.” For the purpose of the present analysis, this variable was coded into two levels with 1 = “Lives with both biological parents” and 0 = “Lives in different circumstances.” Mother education was measured with the question “What is the highest level of school your mother has completed” and responded to on a five‐category ordinal scale ranging from 1 = “Less than high school diploma” to 5 = College graduate.” For the purpose of the present analysis the variable was dichotomized as 1 = “college graduate or higher” and 0 = “less than college graduate.” Finally, comparative family financial status was measured with the question “How well off do you think your family is compared to other families?” Responses ranged from 1 = “much worse off” to 7 = “much better off.”

### 2.5 Analyses

All analyses were conducted in SAS 9.4. Data were treated with pairwise deletion for missing values unless otherwise specified. Descriptive statistics included frequencies and percentages for categorical data and means and standard deviations for continuous data. Matched pair t‐tests for continuous data, and McNemar matched pair tests for categorical data were conducted between Wave 1 and Wave 3 analyses (see Table 1).

**Table 1:** Descriptive statistics for all study variables at Wave 1 and Wave 3.

	Categorical variables (%)		
	Wave 1	Wave 3	McNemar's *p* value
Lives with both parents			.93
Yes	48.2	46.7	
No	51.8	53.4	
Gender			<.0001
Boy	46.2	48.0	
Girl	51.2	45.4	
Other	2.6	6.6	
Mother college degree			<.001
Yes	19.0	21.2	
No	81.0	78.8	
Caffeine			.17
0 mg/day	13.6	12.5	
1–100 mg/day	27.7	24.6	
>100 mg/day	58.6	62.9	

	Continuous variables (mean, SD)		
	Wave 1	Wave 3	Paired *t* test *p* value
Family finance	3.06 (1.36)	2.98 (1.34)	.21
School connectedness	19.62 (3.58)	18.50 (4.31)	<.0001
Supportive adults in school	20.98 (3.96)	19.39 (4.64)	<.0001
Primary caregiver support	13.21 (2.43)	12.64 (2.80)	<.0001
Self‐control	31.12 (6.15)	30.97 (6.42)	.11
Problem behavior	34.31 (11.25)	34.62 (11.43)	.12

Abbreviation: SD, standard deviation.

Linear mixed models (LMM) were conducted on the two key outcomes of interest: problem behavior and self‐control. Two models were conducted for both sets of outcomes: a covariate‐only model (Model 1) including time and all covariates of interest, and then a full model (Model 2) including caffeine as the primary exposure variable. Different LMM covariance structures accounting for the nonindependence in the data due to individual correlations over time were tested, including random and repeated effects and time as both categorical and continuous variables. The best fitting covariance structure was selected via Akaike information criterion (AIC) criteria, and the same structure was applied to all outcomes. The best‐fitting model included a random effect of intercept only, and time was included as a categorical variable. All LMM assumptions were tested and found to be satisfactory.

Linear mixed models effectively use all available data to estimate change over time via Restricted Maximum Likelihood (REML) estimation (Lee & Shang, 2022), utilizing all available participant data regardless of whether a timepoint was missing, and is a preferred method to last‐observation‐carried‐forward methods often used in intent‐to‐treat analyses (Lachin, 2016). Degrees of freedom were adjusted for repeated measures using the Kenward and Roger correction (Kenward & Roger, 1997; Luke, 2017). Fixed‐effect estimates for the categorical time variable with Wave 1 as the referent in addition to all covariates are presented for the primary models for both outcomes, with the second set of models also reporting fixed effect estimates for caffeine use with no use as the referent category. Interactions between caffeine and time were explored and were not statistically significant, and therefore were not included in the final models (i.e., caffeine use is considered stable over time). An *α* level of .05 was chosen as the indicator of statistical significance for all analyses.

## 3 RESULTS

Table 1 includes descriptive statistics for all study variables at Wave 1 and Wave 3. As shown, approximately 28% of students reported using less than 100 mg of caffeine per day and approximately 59% reported using >100 mg per day, with the daily amount of caffeine consumed being largely stable over the observed time period (*p* = .17). Both teacher‐reported dependent variables (*p* > .05) were also stable over time. On the other hand, perception of school connectedness, support from adults in schools, and primary caregiver support decreased over time (*p* < .01).

Table 2 includes results from the LMMs predicting teacher‐observed self‐control. In Model 1, demographic and control variables were entered, and those variables were included in Model 2 together with the two dichotomized caffeine variables. For Model 1, time was not statistically significant, in line with nonsignificant bivariate change in the outcome shown in Table 1. Participants who reported living with both biological parents, with a mother possessing at least a college degree, and who identify as girls, scored significantly higher on teacher‐reported self‐control than boys/other gender and those living in different family circumstances including a mother without a college degree (*p* < .01). The variables of other gender and family finance were not statistically related to the outcome in Model 1. Of the other independent variables, both school connectedness (estimates [Est.] = 0.13) and supportive adults in school (Est. = 0.08) were positively related to self‐control, but primary caregiver support was not (*p* = .21). When caffeine was added to the equation in Model 2 all previous variable relationships were maintained in terms of statistical significance (*p* < .05), while model fits improved based on the AIC criteria. Daily caffeine consumption of <100 mg/day relative to 0 mg/day was not statistically related to the outcome, but daily caffeine consumption of >100 mg/day was inversely related to teacher‐observed self‐control by almost one point on the 36‐range measure (Est. = −.98, *p* < .01) compared to no caffeine use.

**Table 2:** Linear mixed models for the outcome of teacher‐observed self‐control.

	Model 1		Model 2	
	Est. (SE)	*p*	Est. (SE)	*p*
Time	0.043 (0.20)	.830	0.080 (0.20)	.688
Family structure	1.223 (0.23)	<.001	1.180 (0.23)	<.001
Gender (girl vs. boy)	1.455 (0.23)	<.001	1.468 (0.23)	<.001
Gender (other vs. boy)	0.752 (0.51)	.143	0.821 (0.51)	.109
Mother's education	1.180 (0.27)	<.001	1.073 (0.27)	<.001
Family finance	0.037 (0.08)	.650	0.040 (0.08)	.627
School connectedness	0.126 (0.04)	.002	0.125 (0.04)	.002
Supportive adults in school	0.083 (0.04)	.027	0.075 (0.04)	.045
Primary caregiver support	0.056 (0.04)	.208	0.050 (0.04)	.257
Caffeine (<100 mg/day vs. 0)			0.062 (0.36)	.865
Caffeine (>100 mg/day vs. 0)			−0.977 (0.34)	.004
AIC	17,327.6		17,307.9	

Abbreviations: AIC, Akaike information criterion; Est., estimates; SE, standard error.

Table 3 includes results from the LMMs predicting teacher‐observed problem behavior. Similar to the models with self‐control, time was not statistically significant in either Model 1 or 2, in line with the nonsignificant bivariate trend in the outcome shown in Table 1. On the other hand, family structure (living with both biological parents), identifying as a girl or other gender compared to boys, and having a mother with a college education were associated with significantly lower teacher‐observed problem behavior scores in both Models 1 and 2 (*p* < .01). Conversely, family financial status was not statistically related to problem behavior in either model. However, school connectedness, supportive adults in school, and primary caregiver support, were all inversely related to the outcome in both Model 1 (Est. = −0.27, −0.19, −0.19, respectively, *p* < .05) and Model 2 (Est.= −0.27, −0.17, −0.18, respectively, *p* < .05). Again, when caffeine was added to the equation in Model 2, all previous variable relationships were maintained in terms of statistical significance while model fits improved based on the AIC criteria. Daily caffeine consumption of <100 mg/day compared to no caffeine use was not statistically related to the outcome. By contrast, daily caffeine consumption of >100 mg/day was positively related to teacher‐observed problem behavior by just over three points on the 60‐range measure (Est. = 0.3.02, *p* < .01) compared to no caffeine use.

**Table 3:** Linear mixed models for the outcome of teacher‐observed problem‐behavior.

	Model 1		Model 2	
	Est (SE)	*p*	Est (SE)	*p*
Time	0.052 (0.35)	.883	−0.044 (0.35)	.902
Family structure	−3.122 (0.42)	<.001	−3.018 (0.42)	<.001
Gender (girl)	−3.833 (0.44)	<.001	−3.856 (0.43)	<.001
Gender (other)	−4.167 (0.94)	<.001	−4.341 (0.93)	<.001
Mother's education	−2.308 (0.50)	<.001	−2.042 (0.50)	<.001
Family finance	0.161 (0.15)	.283	0.151 (0.15)	.313
School connectedness	−0.270 (0.07)	<.001	−0.265 (0.07)	<.001
Supportive adults in school	−0.185 (0.07)	.007	−0.166 (0.07)	.015
Primary caregiver support	−0.190 (0.08)	.019	−0.176 (0.08)	.029
Caffeine (<100 mg/day)			0.455 (0.66)	.489
Caffeine (>100 mg/day)			3.020 (0.61)	<.001
AIC	20,649.5		20,603.7	

Abbreviations: AIC, Akaike information criterion; Est, estimates; SE, standard error.

## 4 DISCUSSION

To our knowledge, the present study is the first to examine the longitudinal relationship between daily caffeine consumption by middle‐school pupils and teacher‐observed behavior. Specifically, the study examined associations between pupil self‐reported daily caffeine consumption and teacher‐reported student self‐control and problem behavior in 20 West Virginia middle schools. Using survey questions validated in several previous studies (James et al., 2011; Kristjansson, Sigfusdottir, Mann, & James, 2014; Kristjansson et al., 2015, 2018), caffeine intake was operationalized as milligrams of caffeine consumed per day in three categories: no use, <100 mg per day, and >100 mg per day. Compared to no caffeine use, linear mixed models that controlled for demographic and socioeconomic status factors, school connectedness, and social support by adults in school and at home, revealed a robust relationship between caffeine use of >100 mg per day and teacher‐observed self‐control (Est. = −0.98) and problem‐behavior (Est. = 3.02).

Findings from both the present and previous studies by our group indicate that the majority of adolescents regularly consume caffeine in amounts sufficient to produce negative acute effects and recurring withdrawal symptoms during daily periods of nonconsumption. Considering the relatively recent introduction of highly concentrated caffeine products (e.g., caffeine “shots”) and large‐container energy drinks, which are commonly marketed directly at children and youth, our findings should give rise to concerns about the high prevalence of routine caffeine consumption by adolescents, especially when consumption exceeds the comparatively modest level of <100 mg/day. Compared to adults, smaller average body size and lighter weight may render adolescents particularly vulnerable to the acute negative effects of caffeine as well as daily cycles of withdrawal symptoms (Temple, 2019). This, in turn, may exacerbate adolescent behavioral vulnerability to caffeine with respect to potential serious long‐term consequences. As alluded above, diverse direct and indirect causal mechanisms are likely to be responsible for many caffeine‐associated negative behavioral and psychosocial effects among children and youth. In the present context, it is noteworthy that the negative impact of adolescent caffeine use on sleep duration and quality is well established (James et al., 2011) and studies have found a positive association between adolescent caffeine consumption and daytime sleepiness (Kristjansson et al., 2011). Chronic inadequate or poor sleep presumably will negatively impact mood and self‐control in students. Moreover, shared variation between caffeine withdrawal symptoms, risk‐taking tendencies (Kristjansson et al., 2018), and/or sensation‐seeking character (Temple, 2009) may represent a logical line of reasoning for the teacher observations of greater levels of Problem Behavior and lower Self‐Control among students who reported using +100 mg of caffeine per day.

With regard to implications for policy and practice, our findings of inverse associations between adolescent caffeine consumption and teacher‐observed adolescent self‐control and positive association with problem‐behavior do not augur well for long‐term well‐being and success among young caffeine consumers. Moreover, it is evident that the consumption of high‐concentration and high‐volume caffeine products that are legal and easy to obtain is a well‐established and socially accepted pattern of habitual behavior by adolescents (Temple, 2009). Thus, despite likely benefit from limiting adolescent access to and consumption of caffeine, our findings point to substantial challenges to be overcome in the event of attempts to tackle adolescent caffeine consumption as a public health issue. Not least among diverse challenges, there is the need for the general population, the large majority of whom are habitual caffeine consumers, to acknowledge the serious potential negative consequences of early uptake of the habit. In turn, there are additional diverse challenges to be overcome by attempts that would seek to regulate the complex and interconnected commercial production, marketing, distribution, and eventual consumption of the drug by adolescents and adults alike.

This study has some limitations. First, both the student data and teacher reports were based on self‐reports which may be subject to uncontrolled sources of bias. Second, despite considerable geographic variation between the participating schools, the exclusively West Virginia sample may not be representative of other areas. Third, although our caffeine measure has been applied in numerous studies and found to be reliable across multiple sites, it does not include questions about several dietary items that may contain caffeine such as chocolate, candy, and breakfast items. Finally, although we were able to revise our data collection protocols in response to the circumstantial change brought about by the COVID‐19 pandemic and thereby continue to collect survey data with students, we are unable to rule out the potential impact of the COVID‐19 school mitigation efforts on student responses. Notwithstanding those limitations, our study also has some strengths. First, we collected data with a relatively large sample of middle‐school students (i.e., 11–13 years of age), who are much less often than high‐school and college‐aged youth to be included in similar studies. Second, the longitudinal nature of the study enabled us to undertake novel analyses to answer the primary study questions concerning caffeine consumption and teacher‐observed student behavior, while taking account of a several potentially confounding covariates.

## 5 CONCLUSION

We tested the prospective association between daily caffeine consumption and teacher‐observed self‐control and problem behavior in a large sample of middle‐school students in 20 middle schools in West Virginia in the United States. Caffeine was operationalized in two dichotomized variables, daily consumption of <100 mg, and daily consumption of >100 mg versus no daily use. We controlled for several potentially confounding influences on youth behavior, including demographics and social support by primary caregivers and adults in school. For daily users of >100 mg, caffeine was robustly inversely associated with teacher‐observed adolescent self‐control and positively associated with adolescent problem behavior. Given current levels of social acceptance of caffeine use and widespread availability, including high caffeine‐containing products designed specifically for the youth market, our findings suggest the need for intervention to limit child and adolescent accessibility to caffeine‐containing products.

## CONFLICT OF INTEREST STATEMENT

The authors declare no conflict of interest.

## ETHICS STATEMENT

All processes and procedures in this research were reviewed and approved by the respective University Institutional Review Board.

## Data Availability

Deidentified data used in this report is available from the primary author upon request.
